# The differential impact of subjective and objective aspects of social engagement on cardiovascular risk factors

**DOI:** 10.1186/1471-2318-10-81

**Published:** 2010-11-02

**Authors:** Yumiko Kamiya, Brendan Whelan, Virpi Timonen, Rose Anne Kenny

**Affiliations:** 1 Department of Medical Gerontology, Trinity College Dublin, Dublin, Ireland; 2 School of Social Work & Social Policy, Trinity College Dublin, Dublin, Ireland

## Abstract

**Background:**

This article provides new insights into the impact of social engagement on CVD risk factors in older adults. We hypothesized that objective (social participation, social ties and marital status) and subjective (emotional support) aspects of social engagement are independently associated with objective measures of cardiovascular risk.

**Methods:**

Data from the English Longitudinal Study on Ageing (ELSA) were analyzed. The effects of social participation, social ties, marital status, and emotional support on hypertension, obesity, high sensitivity C-reactive protein, and fibrinogen were estimated by logistic regression controlling for age, sex, education, physical function, depression, cardiovascular disease, other chronic diseases, physical activity, and smoking.

**Results:**

Social participation is a consistent predictor of low risk for four risk factors, even after controlling for a wide range of covariates. Being married is associated with lower risk for hypertension. Social ties and emotional support are not significantly associated with any of the cardiovascular risk factors.

**Conclusion:**

Our analysis suggests that participation in social activities has a stronger association with CV risk factors than marital status, social ties or emotional support. Different forms of social engagement may therefore have different implications for the biological risk factors involved.

## Background

Cardiovascular disease (CVD) is the leading cause of morbidity and mortality in Europe (WHO, 2005), accounting for over 4.3 million deaths each year [1], a figure that amounts to nearly half (48%) of all deaths in Europe. Overall CVD is estimated to cost the EU economy €192 billion a year, of which €110 billion (57%) is due to health care costs and €82 billion (43%) to productivity losses and informal care [1].

Evidence from the USA prospective studies has suggested that social engagement lowers the incidence of cardiovascular disease and other chronic diseases [2-6]. Social engagement has been shown to have a beneficial effect on several *behaviours *that affect the risk of cardiovascular disease such as smoking [7-9], smoking cessation [10,11], adherence to medical treatment [12], participation in physical activity [13,14] and diet [15,16]. Social engagement also influences health through *psychological *processes. Social contacts can attenuate stressful experiences by helping to solve problems, or by giving a new interpretation of adverse events [17], thereby buffering the harmful effects of stress [7,18,19]. Social engagement also modulates cardiovascular reactivity [17] via reduced sympathetic nervous system activity and/or stress-related hormonal activity. Social engagement which attenuates the neuro-endocrine stress response [7,17], influences one's emotional state, giving a sense of purpose, meaning and belonging and reducing the intensity and duration of negative affective states [20].

Few studies have examined the possible differential impact of aspects of social engagement and consequently little is known about which components of social engagement are most protective for cardiovascular disease [21]. Disciplinary differences in research approaches contributed to this outcome. While social scientists have focused on "objective" aspects of relationships such as marital status [22], social networks [2], contact with network members, and participation in social activities [23], psychologists have focused on subjective aspects of social relationships such as perceived emotional support and loneliness [24]. In addition, data limitations have encouraged researchers to focus on either subjective or objective aspects of social engagement [25].

In a recent meta-analysis, the protective effects of distinct types of social support on the incidence and prognosis of coronary heart disease (CHD) were mixed. While there was evidence that emotional support was protective against the prevalence and disease progression of CHD, the results were unclear for structural support (defined as number of contacts, membership of community groups and marital status) [26].

The aim of this paper is to make a contribution to the literature by examining the effects of social engagement on CV risk factors. Our use of direct measures of health instead of self-perceived (self-reported) health, is a further improvement from previous studies, because direct measures reduce the likelihood of reporting bias errors [27].

In this article, 'social engagement' refers to a combination of objective and subjective measures of the salient aspects of people's 'social' existence. The objective measures are defined by connectedness to other individuals (the number of children, friends and relatives whom the respondent feels are close to him/her) and participation in social groups (affiliation to or membership in religious, voluntary, political, and social associations or activities). The first, we refer as "social ties", and the second as "social participation". The subjective measures comprise of perceptions of available emotional support from spouse, children, relatives and friends. These components are based on previous studies of older individuals [28]. We hypothesized that objective (social participation, social ties and marital status) and subjective (emotional support) aspects of social engagement are independently associated with objective measures of CV risk.

The risk factors examined here have well-established associations with cardiovascular diseases: hypertension, obesity and two inflammatory markers - fibrinogen, and high sensitivity C-reactive protein (hsCRP). Epidemiological studies have shown that both systolic (SBP) and diastolic blood pressure (DBP) are important cardiovascular risk factors [29]. Obesity, measured by BMI, is an independent predictor of coronary heart disease [29,30]. Both high sensitivity CRP and fibrinogen are acute phase inflammation reactants. Prospective studies and meta-analysis have demonstrated that hsCRP [31-34] and fibrinogen [35,36] are strong independent predictors of the risk of cardiovascular disease in healthy individuals and in those with pre-existing CVD. Specifically, hsCRP is a predictor of frailty [37], a risk factor for the development of arterial fibrillation [38], ischemic stroke [39], and diabetes [40]. Fibrinogen is involved in thrombogenesis and in the stimulation of atherogenenic cell proliferation; elevated levels of fibrinogen are associated with coronary disease and stroke [41,42].

## Methods

### Design/participants

The English Longitudinal Study of Ageing (ELSA) is an ongoing panel study of a nationally representative sample of the English population living in households. The original ELSA cohort consists of men and women born on or before 29 February 1952. The sample was drawn from households that had participated in the Health Survey for England (HSE) in 1998, 1999, and 2001. For the present analyses, data from the first wave (baseline, 2002-2003) and second wave (2004-2005) were used. The first and second wave involved a face-to-face interview. The second wave also included a clinical assessment by a nurse. This is the only wave containing the objective health assessment and blood analysis data that is currently available for public use. Overall, 10,770 participated in wave 1 (response rate 65.7%). Of these, 8,688 people participated in wave 2 (82%) and 7,433 participants were willing to have a nurse visit. Of these, 6,649 people consented to a blood sample.

Valid blood samples are available for 5,884 people of which 4,432 (75%) were fasting. Fasting blood samples were taken for most respondents under the age of 80 except people who had known diabetes and were on treatment, or whose heath the nurse was concerned about. Subjects were considered to have fasted if they had not had food or drink for a minimum of 5 hours prior to the blood test. However, there was no considerable differences in the mean (Chi square test) for those who had fasted (fibrinogen mean = 3.27 and SD = 0.0147; hsCRP mean = 2.52 and SD = 0.045) and for those who had not fasted (fibrinogen mean = 3.33 and SD = 0.019; hsCRP mean = 2.703 and SD = 0.058). Because this is a prospective population study (n = 8,688), due to the length of fieldwork (July 2004- July 2005) and the overall fieldwork capacity, it was not possible to allow for circadian effects by restricting nurses to, for example, morning-only appointments. While hsCRP has no circadian or seasonal variation [43,44], the literature shows that for fibrinogen the proportion of variation attributed to the diurnal, seasonal, and processing effects was only 2% [45].

Biological data were missing for participants who did not consent to give blood (participants recently had a blood test, disliked needles or had previous difficulty with venipuncture, n = 993), were ineligible (participants with clotting and bleeding disorders, or taking anti-coagulant medication, n = 351) or for whom attempts to obtain a blood sample were unsuccessful (incomplete/partial sample taken, insufficient blood for test or no suitable/palpable vein/collapsed vein, n = 360). The analysis of the blood data was carried out in the Royal Victoria Infirmary (Newcastle-upon-Tyne, UK). Both the HSE and ELSA employed the same laboratory and the same guidelines and protocols for the blood analysis. Detailed information on the technicalities of the blood analysis, the internal control, and the external quality assessment are available in the 2004 HSE technical report [46]. Blood samples were analyzed for hsCRP and fibrinogen. Analysis of hsCRP levels from serum was performed using the N Latex high sensitivity CRP mono immunoassay on the Behring Nephelometer II analyser. The limit of detection was 0.17 mg/l and the coefficient of variation (CV) was less than 6% for this assay. Fibrinogen levels were determined using the Organon Teknika MDA 180 analyser, using a modification of the Clauss thrombin clotting method, with a CV of less than 10%. These are acceptable limits in a large population based sample. Blood pressure was recorded as the average of three seated blood pressure readings (Omron HEM-907 blood pressure monitor).

In comparison with the overall sample (Chi square tested), the sub-group of people who donated blood were younger (mean age 66 years old vs. 69 years), had a lower prevalence of morbidity (39.1 vs. 26.2%), better self-reported health (75.3% vs. 60.5%) and better health behaviours including lower rates of smoking (18.9% vs. 16.9%) and were less sedentary (reported no frequency of physical activity 6.5% vs. 15%). However, social engagement variables were not different between respondents who agreed to blood donation and those for whom blood was not available for analysis.

In order to account for non-response to blood sample, weights were used which aimed at reducing any bias arising from differential non-response between completion of the nurse visit and giving a blood sample [47]. Using these weights allows correction for non-response at interview, nurse visit, and in providing a non-fasting blood sample.

The final weights used thus incorporate adjustment for four levels of attrition/non-response: 1) from initial sample (HSE) to wave 1; 2) from wave 1 to wave 2; 3) from interview at wave 2 to the nurse visit; and 4) non-response to blood donation. These weights attenuate the potential selection biases due to attrition at different stages and should ensure that the weighted data will be representative of the English population living in the community who are over 50 years-old [48]. Details on the calculations of weights are presented in the ELSA technical report [48].

### Measurements

#### Cardiovascular risk markers

Cardiovascular risk variables were dichotomized. *Hypertension *is defined as SBP and DBP ≥ 140/90 mmHg [49] or SBP ≥ 140 (isolated systolic hypertension) or using hypertensive medication. *Obesity *was measured by body mass index (BMI), which was dichotomized by means of a cut-off point of ≥ 30 kg/m2. For both hsCRP and fibrinogen [50], clinical cut-off points were used; *hsCRP *>3.0 mg/l was interpreted as high risk as it corresponds approximately to the highest tertile of hsCRP in the adult population [50]. Cases with hsCRP higher than 10 mg/L were excluded from the hsCRP analysis because such levels reflect an acute infection or inflammation other than those due to cardiovascular disease [51]. For *fibrinogen*, values >4.0 g/L are a valid clinical cut-off and correspond to the highest quartile concentration in the adult population [52].

#### Social engagement

Three different dimensions of social engagement were examined. *Social participation *was measured as a count of seven activities in which the respondent reported *current *membership or participation in any of a list of groups and associations divided into: (1) political, trade union or environmental group; (2) tenants' groups, residents' groups or neighbourhood watch; (3) church or other religious organization; (4) charitable associations; (5) an education, arts or music group or evening class; (6) social club (e.g. Rotary Club, elderly lunch group, women's group); and (7) any other organisations, clubs or societies. The social participation raw score therefore ranges from 0 to 7. This was standardized as a Z-score. Scores range from -0.899 to 4.202 with weighted mean of -0.087 (SD = 0.953). Higher scores indicate greater social participation. *Social ties *were measured by a count of the number of children, relatives and friends the participant felt close to ("How many of your children/relatives/friends would you say you have a close relationship with?"). The final score was standardized and its value was averaged across the ties that were relevant for a given respondent. Scores range from -1.256 to 1.533 with weighted mean of -0.090 (SD = 0.301). *Emotional support *from spouse, children, relatives and friends was measured by the following three questions: a) How much respondents feel their spouse/partner (children/relatives/friends) understand(s) their feelings; b) How much respondents can rely on their spouse/partner (children/relatives/friends) if they have a serious problem; and c) How much respondents can open up to their spouse/partner (children/relatives/friends) if they need to talk. The responses for each item range from 0 (not at all) to 3 (a lot). Responses to all twelve questions were added up to a summary score (Cronbach's alpha was 0.88). The emotional support scale was constructed by standardizing and then averaging across the relevant item scores. Standardized scores range from -2.409 to 0.1838 with weighted mean of -0.174 (SD = 0.554). *Marital status *was dichotomized as married (or cohabiting) and not married (never married, separated or divorced, and widowed).

Demographic and socio-economic variables included age (in years), age squared, sex (male as reference category), and education measured as the highest qualification participants obtained, and categorized into four groups (no education, primary, secondary and tertiary level).

#### Health behaviours

Smoking was coded as never smoked and ever smoked (ex-smoker or current smoker). Self-reported physical exercise was classified as none, light, moderate and vigorous activity at least once a week.

#### Co-morbidity

Physical function was assessed by dichotomizing the Activities of Daily Living into "0 ADL", reporting no ADL difficulties and ""≥ 1 ADL", reporting one or more ADL difficulties. Known cardiovascular disease was assessed by self-reported angina, myocardial infarction, diabetes, stroke, heart failure, heart murmur, abnormal heart rhythm, and ischaemic heart disease. Other major chronic diseases include self-reported chronic lung disease, asthma, arthritis, osteoporosis, cancer, and Parkinson's disease. These variables are dichotomized into "0 conditions" and "≥1 conditions". Depression was measured by the 8-item Center for Epidemiologic Studies Depression Scale (CES-D) with a cut-off point of 3 or more depressive symptoms [53] (<3 = non depressive, ≥3 = depressive).

### Statistical Analysis

We used Spearman's rho to test for independence among social engagement variables. The correlations between social participation and social ties (r = 0.15, p < 0.001), and social participation and emotional support (r = 0.19, p < 0.001) were weak in strength. Correlation between social ties and emotional support was moderate in strength (r = 0.32, p < 0.001). Correlations between marital status and emotional support was moderate in strength (r = 0.29, p < 0.001), while correlation between marital status and social participation (r = 0.04, p < 0.001), and marital status and social ties were weak in strength (r = 0.05, p < 0.001). Therefore, objective and subjective measurements were not strongly correlated and, since they are conceptually distinct, we expect that these four dimensions would have independent associations with cardiovascular risk markers.

Using logistic regression, each cardiovascular risk factor (hypertension, BMI, fibrinogen, and hsCRP) at wave 2 was regressed on the complete set of social engagement variables at baseline (social participation, social ties, marital status and emotional support). The independent effect of each of the social engagement variables was examined by putting all the social engagement variables and confounding factors (age, gender, education, co-morbidity and behavioral risk factors) into a single model. Seasonality was adjusted for fibrinogen. Age squared was used to test for the non-linear relationship between age and the outcome variables, and an interaction term between sex and age was also included. The result of Goodness-of-fit tests (such as Akaike Information Criterion- AIC and Bayesian Information Criterion-BIC) supported the model with interaction and quadratic term over the nested model (with no interaction and quadratic term). Data were weighted for panel attrition.

## Results

Table 1 presents characteristics of the sample at baseline. The sample was composed of 53.4% women and 46.6% men. The median age was 65.4 years (66.3 and 64.3 years for women and men respectively). Sixty seven percent were married, and 40.5% had a primary education. Thirty-seven percent reported no social participation, and 15.1% of the respondents reported that they did not have children, relatives or friends that they felt close to. Nighteen percent reported having cardiovascular morbidity, 39.7% reported having non-cardiovascular chronic conditions, 29.9% reported difficulties with at least one ADL, and 22.9% had depressive symptoms. Approximately 8% were relatively sedentary, reporting no physical activity and 17.5% were smokers.

**Table 1:** Descriptive characteristics of the sample at baseline and CV risk factors at wave 2

Variable	Percent/Mean (SD)
**Social engagement**	
Social participation	1.23 (1.37)
Social ties	6.42 (5.22)
Emotional support	2.82 (3.51)
**Demographic and socio-economic variables**	
Age (%)	
50-60 years	37.27
60-70 years	28.42
70-80 years	22.49
80+ years	11.82
Mean Age	65.35 (10.60)
Currently married (%)	66.70
Female (%)	53.70
Education: levels of education attained	
tertiary	22.91
secondary	27.87
primary	40.50
no education	8.72
**Co-morbidity**	
Have Depression (8 items CES-D)	22.90
No chronic disease*	60.21
No CVD**	81.16
No limitations with ADLs	70.10
**Health Behaviours**	
Never smoked	82.46
Physical activity	
none	8.24
light	15.75
moderate	48.65
vigorous	27.36
**Cardiovascular variables**	
Systolic and diastolic blood pressure	
Mean systolic BP (mmHg)	135.4 (19.0)
Mean diastolic BP (mmHg)	74.9 (11.3)
Hypertension (%)	38.78
Body Mass Index (>30 kg/m2)	28.90
Mean hsCRP (mg/l)	2.55 (2.16)
Mean Fibrinogen (g/l)	3.2 (0.7)

Note: For hsCRP and fibrinogen, n = 5884. For all other variables, n = 7433

*chronic lung diasese, arthritis, osteoporosis, cancer, parkinson's disease

**angina, diabetes, myocardial infection, stroke, heart failure, heart murmur, abnormal heart rhythm, valvular heart disease, ischaemic heart disease

Tables 2 and 3 shows the odds ratios (OR) and 95% confidence intervals (CIs) from logistic regression. The results of these tables show whether baseline social engagement predicted the cardiovascular risk factors at follow-up. First, we fitted a model controlling for age, gender, education and co-morbidity (physical function, depression, cardiovascular and chronic disease). In addition, behavioral risk factors (smoking, and physical activity) known as confounders or mediators were adjusted in model 2.

**Table 2:** Logistic regression models for hsCRP and fibrinogen

	hsCRP		fibrinogen	
	
	Model 1	Model 2	Model 1	Model 2
	OR(95% CI)	OR(95% CI)	OR(95% CI)	OR(95% CI)
Age	1.156	1.186	1.202	1.222
	(1.082 - 1.234)**	(1.109 - 1.268)**	(1.129 - 1.279)**	( 1.148-1.303)**
Age squared	0.999	0.999	0.999	0.999
	(0.999 - 1.000)**	(0.998 - 0.999)**	(0.998 - 0.999)**	(0.998 - 0.999)**
Female	2.923	3.025	1.772	1.765
	(1.309 - 6.527)**	(1.338 - 6.843)**	(0.795-3.953)	(0.781-3.993)
Age*sex	0.986	0.985	0.993	0.994
	(0.974 - 0.998)*	(0.974 - 0.997)*	(0.982 - 1.006)	(0.982 - 1.006)
Education	0.884	0.903	0.872	0.892
	(0.825 - 0.947)**	(0.842 - 0.968)**	(0.795- 3.953)**	(0.831-0.957)**
Married	0.908	0.947	0.875	0.927
	(0.791 - 1.043)	(0.823 - 1.091)	(0.765 - 1.001)*	(0.807-1.063)
Social participation	0.902	0.934	0.889	0.929
	(0.846 - 0.962)**	(0.875 - 0.997)*	(0.834 - 0.948)**	(0.871 - 0.992)*
Social ties	0.971	0.97	0.976	0.973
	(0.912 - 1.034)	(0.911 - 1.034)	(0.917-1.038)	(0.914-1.036)
Emotional support	1.025	1.041	0.925	0.95
	(0.877 - 1.199)	(0.885 - 1.223)	(0.801 - 1.067)	(0.818 - 1.102)
ADL	1.422	1.283	1.527	1.402
	(1.231 - 1.642)**	(1.105 - 1.490)**	(1.329 - 1.755)**	(1.213 - 1.621)**
Depression	1.007	0.960	1.115	1.049
	(0.869 - 1.166)	(0.826 - 1.115)	(0.968 - 1.283)*	(0.9081 - 1.212)
CVD	0.983	0.940	1.083	1.041
	(0.838 - 1.153)	(0.799 - 1.106)	(0.929 - 1.263)	(0.890-1.217)
Chronic disease	1.269	1.224	1.118	1.081
	(1.158 - 1.390)**	(1.115 - 1.344)**	(1.024 - 1.221)*	(0.987 - 1.182)
Never smoked		0.643		0.563
		(0.549 - 0.753)**		(0.4832 - 0.655)**
Physical activity		0.851		0.851
		(0.805 - 0.899)**		(0.805 - 0.889)**
Seasonality			1.196	1.190
			( 1.136-1.258)**	( 1.130-1.254)**

95% confidence intervals in parentheses

* significant at 5%; ** significant at 1%

Model 1 controls for age, gender, education, and comorbidity (physical ability, depression, chronic conditions and cardiovascular disease)

Model 2 contains all adjustments from Model 1 with addition of health behaviours

**Table 3:** Logistic regression models for hypertension and BMI

	hypertension		BMI	
	
	Model 1	Model 2	Model 1	Model 2
	OR(95% CI)	OR(95% CI)	OR(95% CI)	OR(95% CI)
Age	1.121	1.114	1.123	1.139
	(1.064 - 1.182)**	(1.056 - 1.175)**	(1.047- 1.203)**	(1.060 - 1.224)**
Age squared	0.999	0.999	0.9989	0.999
	(0.999 - 1.000)**	(0.999 - 1.000)**	(0.998 - 0.999)**	(0.998 - 0.999)**
Female	0.180	0.183	0.5435	0.558
	(0.095 - 0.340)**	(0.096 - 0.347)**	( 0.253 - 1.211)	( 0.257 - 1.211)
Age*sex	1.022	1.021	1.0119	1.011
	(1.012 - 1.032)**	(1.012 - 1.031)**	(1.000 - 1.023)*	(0.999 - 1.023)
Education	0.937	0.933	0.8890	0.887
	(0.887 - 0.990)*	(0.883 - 0.987)*	(0.836 - 0..945)**	(0.833 - 0.944)**
Married	0.859	0.846	1.1386	1.111
	(0.768 - 0.961)**	(0.755 - 0.948)**	(1.005 - 1.289)*	(0.978 - 1.262)
Social participation	0.934	0.930	0.9026	0.897
	(0.888 - 0.982)**	(0.884 - 0.979)**	(0.852 - 0.956)**	(0.846 - 0.952)**
Social ties	0.992	0.991	1.0577	1.061
	(0.945 - 1.042)	(0.943 - 1.041)	(1.002 - 1.116)*	(1.005 - 1.120)
Emotional support	1.067	1.092	0.9474	0.968
	(0.944 - 1.206)	(0.963 - 1.238)	(0.826 - 1.086)	(0.840 - 1.116)
ADL	1.052	1.051	1.6548	1.542
	(0.933 - 1.185)	(0.929 - 1.189)	(1.456 - 1.881)**	(1.350 - 1.762)**
Depression	0.942	0.938	0.9364	0.921
	(0.836 - 1.060)	(0.832 - 1.058)	(0.821 - 1.068)	(0.805 - 1.053)
CVD	0.843	0.832	1.5589	1.479
	(0.743 - 0.957)**	(0.731 - 0.946)**	(1.361 - 1.785)***	(1.288 - 1.699)**
Chronic disease	0.966	0.964	1.2305	1.215
	(0.896 - 1.042)	(0.894 - 1.040)	(1.134 - 1.335)**	(1.118 - 1.320)**
Never smoked		1.033		1.727
		(0.910 - 1.173)		( 1.005 - 1.289)**
Physical activity		0.995		0.839
		(0.953 - 1.039)		(0.799 - 0.882)**

95% confidence intervals in parentheses

* significant at 5%; ** significant at 1%

Model 1 controls for age, gender, education, and comorbidity (physical ability, depression, chronic conditions and cardiovascular disease)

Model 2 contains all adjustments from Model 1 with addition of health behaviours

In model 1, social participation was inversely associated with all four of the CV risk markers (p < 0.05). For example, an increase of one standard deviation in social participation was associated with about 7% lower odds of having hypertension, 11% lower odds of being obese, and 10% and 12% lower odds of having higher levels of hsCRP and fibrinogen, respectively. Being married was inversely associated with hypertension and fibrinogen; being married would reduce the odds of having hypertension by 15%, and the odds of elevated levels of fibrinogen by 16%.

Model 2 assesses the roles of behavioral factors as potential mediators or confounders of the relationship between social engagement and CV risk factors. When behavioural factors are controlled for, the associations between social participation and hypertension and BMI remained unchanged. However, behavioral factors attenuated the results for hsCRP (from 10% to 7%), and for fibrinogen (from 12% to 8%).

We also performed analysis without adjusting for the weights (results upon the request). The results were similar and remained significant for those variables discussed in the main text. In addition, to examine whether social engagement protects against CV risk factors for those with pre-existing CVD, we constructed a version of model 2 with interaction terms. In those who had a pre-existing CVD, social ties had a protective effect by lowering the hsCRP level (OR = 0.84, 95% CI: 0.716-0.995) and emotional support increased the odds of being obese (OR = 1.58; 95% CI: 0.998-2.50). The other dimensions of social engagement such as marital status and social participation were not significant.

## Discussion and Conclusions

Previous studies [5,19,54,55] examining objective aspects of social engagement (i.e. social participation and social ties) found no consistent association between inflammatory markers (hsCRP and fibrinogen) for older and younger women and younger men. The authors recommended that subjective aspects of social engagement (such as emotional support) should be addressed in further studies in order to provide insight into the role of inflammatory markers as biological mediators between social engagement and cardiovascular disease. In addition, the way that the social engagement variable was constructed in the previous studies made it difficult to identify which particular aspects of people's social relationships are associated with cardiovascular disease. This paper addressed this issue by incorporating both objective and subjective measurements. Furthermore, this paper explored how social engagement may protect against cardiovascular disease by examining its association with cardiovascular risk factors.

Results indicate that within the objective measures, behavioral factors may mediate the relationship between social participation and inflammatory markers i.e., the risks of having higher concentrations of inflammatory markers may operate partially through health behaviours. Conversely, for BMI and hypertension, social participation seems to operate distinctly from behavioral factors and is an independent predictor of CV risk markers.

Marriage is associated with hypertension and it is not mediated by behavioral factors. Contrary to what has been suggested in the literature, social ties and emotional support were not significant for CV risk factors [19]. We also tested whether emotional support mediates the relationship between social ties and CV risk factors, but could not find strong evidence of such mediation. Additional analysis was performed (not shown) to include measures of loneliness (only available at wave 2). However, controlling for depression (CES-D minus the loneliness item), the effect of loneliness was not significant.

Successive meta-analysis and prospective studies show evidence that depression is associated with an increased risk of all-cause and cardiovascular death and that this risk is particularly marked in depressive participants with co-morbid CHD [56-59]. However, the association between inflammatory markers and depression have been unclear [60]. In this study, only fibrinogen was modestly associated with depression but not hsCRP. Other studies, such as the Longitudinal Aging Study Amsterdam found similar results, with no association between CRP and depression [61]. Therefore, the mechanisms linking depression to inflammatory markers are still poorly understood. Meta-analysis in the future would help to clarify these contradictory findings. Some limitations of how depressive symptoms were measured in ELSA need to be also considered. The reduced 8 items of CES-D scale assesses depressive symptoms rather than clinical depression. CES-D is therefore not a diagnostic test of depression but poor scores are indicative of possible depression.

A recent study on social participation [62] did not find any consistent association with CV risk factors. However, the study compared the mean scores of the CV risk markers by social participation adjusted only for age and social class. Our study adjusted for various indicators of comorbidity and behavioral factors to reduce the likelihood that the observed relationship between social engagement and CV risk factors was a spurious one.

The findings suggest that participation in social, civic or political activities may have an important effect on lowering CV risk factors. Previous research has found that social participation has an impact on physical and mental health [63-65], survival at older ages [66], and is inversely associated with plasma fibrinogen [67,68]. Participation in political, social or civic activities provides social contacts and gives rise to meaningful social roles which in turn provide a sense of value, belonging and attachment in the community [28]. Social participation may give meaning and purpose to life through the fulfillment of various social roles [69], thus lowering the levels of psychological distress [66]. Social participation may facilitate access to health information and services, and provide access to resources such as information, transportation, and emotional support [21]. It may even exert social control by encouraging health promoting behaviors such as exercise, diet or discouraging health damaging behaviors such as smoking, excessive eating, alcohol consumption and drug abuse [70].

The results also show that marriage was associated with hypertension. The protective relationship between marriage and morbidity/mortality from cardiovascular disease has been established in many studies [71]. Marriage may reduce stress and stress related illness [22], by attenuating the effects of stress on cardiovascular hyper-reactivity and on exaggerated sympathetic nervous system activity [71].

This study has both strengths and limitations. Strengths include a large representative sample of the non-institutionalized older population from which the findings can be generalized, the use of four separate indicators of social engagement, and the careful direct measurement of a range of relevant and previously validated cardiovascular risk factors. The limitations are firstly that causality cannot be inferred from cross-sectional data analysis (necessitated by the fact that availability of health measurement and blood data is currently limited to a single wave of the study). Secondly, despite the fact that we controlled for physical activities and that social participation was not correlated with physical activities, one could argue that social participation is actually measuring physical activities in ways not otherwise controlled for. We tested whether some of the social participation items such as participation in tenants' groups or neighbourhood watch might incorporate some amount of physical activity. A correlation test was carried out on the relationship between physical activities and each type of social participation, but only the item referring to "attending education, arts or music groups or evening class" was weakly correlated (r = 0.127, p < 0.001). The other activities were not correlated. In addition, in model 2 presented in the paper, social engagement and physical activity were entered simultaneously and both variables were significant. This result adds to our confidence that these variables are independent predictors of CV risk factors and the effect of social participation is not due to the impact of physical activity. Third, although we adjusted for an extensive range of health factors, one could argue that social participation is actually measuring health status in ways not otherwise controlled for. Future analyses of forthcoming panel data will allow us to explore this and draw stronger causal inferences from further waves.

This article has explored the relationships between different aspects of social engagement and cardiovascular risk factors. Examination of different dimensions of social engagement suggests that we can identify more precise biological pathways through which social engagement influences cardiovascular disease. The analysis advanced here, it is hoped, will be of relevance to scholars working at the intersection of the social and biomedical sciences, who are seeking to understand the complex interactions between social engagement and CVD.

## Competing interests

The authors declare that they have no competing interests.

## Authors' contributions

YK performed the statistical analysis, interpreted the results and drafted the manuscript. BJW and VT participated in interpretation of the results and drafting the manuscript. RK was responsible for the study interpretation and manuscript write-up. All authors read and approved the final manuscript.

## Pre-publication history

The pre-publication history for this paper can be accessed here:

http://www.biomedcentral.com/1471-2318/10/81/prepub
